# Dietary guideline adherence for gastroesophageal reflux disease

**DOI:** 10.1186/1471-230X-14-144

**Published:** 2014-08-14

**Authors:** Ai Kubo, Gladys Block, Charles P Quesenberry, Patricia Buffler, Douglas A Corley

**Affiliations:** 1 Kaiser Permanente Division of Research, University of California, San Francisco, 2000 Broadway, Oakland, CA 94612, USA; 2 University of California, Berkeley; School of Public Health, Berkeley, CA 94720-7360, USA

**Keywords:** Gastroesophageal reflux, Heartburn, Diet

## Abstract

**Background:**

Gastroesophageal reflux disease (GERD) is the most common gastrointestinal disease, and the cost of health care and lost productivity due to GERD is extremely high. Recently described side effects of long-term acid suppression have increased the interest in nonpharmacologic methods for alleviating GERD symptoms. We aimed to examine whether GERD patients follow recommended dietary guidelines, and if adherence is associated with the severity and frequency of reflux symptoms.

**Methods:**

We conducted a population-based cross-sectional study within the Kaiser Permanente Northern California population, comparing 317 GERD patients to 182 asymptomatic population controls. All analyses adjusted for smoking and education.

**Results:**

GERD patients, even those with moderate to severe symptoms or frequent symptoms, were as likely to consume tomato products and large portion meals as GERD-free controls and were even more likely to consume soft drinks and tea [odds ratio (OR) = 2.01 95% confidence interval (CI) 1.12-3.61; OR = 2.63 95% CI 1.24-5.59, respectively] and eat fried foods and high fat diet. The only reflux-triggering foods GERD patients were less likely to consume were citrus and alcohol [OR = 0.59; 95% CI: 0.35-0.97 for citrus; OR = 0.41 95% CI 0.19-0.87 for 1 + drink/day of alcohol]. The associations were similar when we excluded users of proton pump inhibitors.

**Conclusions:**

GERD patients consume many putative GERD causing foods as frequently or even more frequently than asymptomatic patients despite reporting symptoms. These findings suggest that, if dietary modification is effective in reducing GERD, substantial opportunities for nonpharmacologic interventions exist for many GERD patients.

## Background

Gastroesophageal reflux disease (GERD) is one of the most common illnesses in the United States. The prevalence of diagnosed GERD cases is estimated to be 13-19% worldwide [1] and about 25% of the Western population suffers from heartburn or acid regurgitation at least once per month; 12% have symptoms at least weekly and 5% experience heartburn daily [2]. Symptoms associated with GERD include heartburn, acid regurgitation, dysphagia (difficulty in swallowing), and chest pain, as well as extraesophageal manifestations such as nausea, chronic cough, asthma, and hoarseness [3,4].

The financial burden of disease from GERD is high; much of this is for anti-acid medications. The cost of health care and lost productivity due to GERD is estimated at over $24 billion annually in the US alone [5-7], 60% of which is used for medication [8]. The annual average medical costs and services for patients with GERD may be twice that of the non-GERD population due to additional outpatient visits, hospitalizations, emergency department utilization, and pharmacy costs [5]. In addition, the quality of life in GERD patients is also compromised: the impact of GERD on quality of life measures is similar to that of other chronic diseases such as diabetes, heart failure, or ischemic heart disease [9].

Newly recognized potential adverse effects of acid suppression have heightened the interest in nonpharmacologic approaches for GERD treatment in persons with nonerosive GERD. Although results conflict somewhat between studies, acid suppression has been recently associated with decreased absorption of dietary calcium and calcium supplements [10,11], an increased risk of hip fractures [12-14], food borne infections, and an increased risk of clostridium difficile infection [15,16]. These findings led to a recent modification in the labeling of proton pump inhibitors to include concerns about an increased risk of hip fracture and a search for methods to decrease GERD patients’ reliance on pharmacologic methods of acid suppression.

Dietary modification is a proposed first-line therapy for patients with GERD. The National Institutes of Health and the American College of Gastroenterology recommend that patients with GERD reduce their intakes of total fat, chocolate, alcohol, citrus and tomato products, coffee, tea, and large meals, and implement other lifestyle changes such as stopping smoking and weight reduction [17,18]. However, no previous population-based study has evaluated what proportions of GERD patients actually follow these dietary guidelines. Therefore, we evaluated, within a large community-based population, the associations between a GERD diagnosis, reflux symptoms and dietary intake. We hypothesized that patients with more severe or frequent symptoms were less likely to adhere to the dietary modification recommendations.

## Methods

### Study population

We performed an analysis of a case–control study of Barrett’s esophagus; the study recruited members of the Kaiser Permanente, Northern California (KPNC) population, an integrated health services delivery organization, between 2002 and 2005. The KPNC membership contains approximately 3.3 million persons whose demographics closely approximate the underlying census population of Northern California [17,18]. The study included Barrett’s esophagus patients as cases and two sex and age-matched control groups: GERD patients and population controls. The details of this original study were described previously in detail [19]. The present analysis contrasts the GERD and population control groups. The study and analyses were approved by the KPNC institutional review board and were performed in accordance with the Declaration of Helsinki. Informed consent was obtained.

### GERD patients

GERD patients consisted of 317 eligible KPNC members who were selected from among all individuals with a physician-assigned GERD diagnosis (e.g. ICD-9 codes 530.11 reflux esophagitis and 530.81 gastroesophageal reflux) who also had a prescription for at least 90 days supply of anti-secretory medications and no diagnosis of Barrett’s esophagus (any visible columnar metaplasia) from an esophagogastroduodenoscopy close to the index date. Endoscopy and pathology reports were manually reviewed. The validity of identifying GERD patients using encounter and pharmacy databases was previously evaluated in a 100,000 member managed care organization [20]. All patients in the current study also ultimately received questionnaires regarding GERD symptoms and actual medication use.

Exposure to anti-secretory medications utilized a pharmacy database with information on prescription type, frequency of dispensing, and number of pills dispensed. We defined anti-secretory medications (e.g., histamine-2 receptor antagonists (H2 blocker) or proton pump inhibitors (PPI)) using data from the year prior to control selection (the reference dates).

### Population controls

Population controls were randomly selected from among members of the entire KPNC membership at the time the Barrett’s esophagus cases were identified. Individuals with Barrett's esophagus were excluded. For the current study, we also excluded population controls who, upon interview, reported heartburn or acid regurgitation once a month or more.

### Measurements of dietary intake

All study subjects completed: an in-person interview (most commonly at the subject’s home) of GERD symptoms, medication use, medical history, diet, tobacco use and alcohol use; phlebotomy; and anthropometric measurements.

We assessed dietary intake using a validated 110-item food frequency questionnaire (FFQ) [21-24]. This questionnaire estimates average daily nutrient intake based on questions about frequency and portion size of a given food and usual eating habits for the year before the index date [25]. Dietary data were translated to daily intakes of specific nutrients using NutritionQuest (Berkeley, CA), and the raw data were used to estimate weekly consumption of particular food items. We excluded subjects with over 20 missing food items or with extremely high (>6000 kcal/day) or low (<400 kcal/day) total daily energy intakes.

We evaluated the associations between consumption of commonly discussed “reflux-triggering” dietary factors based on recommendations from the American College of Gastroenterology [17], National Institute of Health [18], and food items that have been studied in previous clinical research of GERD [26,27]. Among those food items, data were available in our questionnaire for: coffee, tea, soft drinks, alcohol (total, wine, beer, and liquor), citrus, tomato products, total fat, fried foods, and large portion meals. “Soft drink” consumption was used as the proxy for “carbonated drinks” in the data; the questionnaire did not include diet sodas. Coffee and tea consumption did not distinguish between caffeinated or decaffeinated types, but excluded herbal tea. The food items and nutrients included total fat; fried foods (e.g., French fries, fried chicken, fish, and doughnuts); tomato products (e.g., fresh tomatoes, canned tomatoes, and tomato juice); and citrus (fruits and juice). The measurement units were mostly “servings per week”, except for fat (grams/day), coffee and tea (cups/day) and alcohol (drinks/day). The term ‘drink’ for alcohol was defined as: wine (7-8 oz), beer (12 ounces), liquor (one shot). Portion size reporting was facilitated by pictures depicting small (1/4 cup), medium (1/2 cup), large (1 cup), and extra-large (2 cups) servings. In this study, each person was scored on the proportion of foods that were reported as large or extra large. For instance, if a person indicated that half of what he/she consumed was in large or extra-large portions (1–2 cups), the person was considered to consume 50% of the meal in large portion. On the other hand, if a person always consumed small or medium portion meals (1/4-1/2cup), the person was categorized to consume 0% of the meal in large portion.

We constructed tertile categories for items with a normal distribution, such as fat intake and portion size. For most other food items, the distributions were skewed with large number of subjects consuming none of the item. For those items, intakes were categorized as follows: zero intake (referent), low intake, and high intake. Tertile cutoff points were chosen *a priori* (prior to analysis) to create relatively even distributions of population controls.

### Measurements of GERD symptoms

GERD symptoms were defined as either heartburn (a burning pain or discomfort behind the breastbone) or acid regurgitation (a bitter or sour-tasting fluid coming up into the throat or mouth) using a validated questionnaire [28]. Symptom severity was recorded as either mild (could be ignored), moderate (could not be ignored, but didn’t affect lifestyle), severe (could not be ignored and did affect lifestyle), or very severe (markedly affected lifestyle). Symptom frequency was coded as: less than once a month, less than once a week (but more than once a month), once or more a week, or daily. For the analysis of severity, we compared individuals who had moderate to severe symptoms (with at least monthly frequency) to asymptomatic population controls, and excluded individuals with mild severity (N = 75). For frequency analysis, we compared two categories (GERD symptoms greater than several times a week; more than once a month to once a week) to asymptomatic population controls.

### Statistical analysis

We evaluated whether GERD was associated with the intake of certain beverages, nutrients, or food items using unconditional logistic regression to calculate odds ratios (ORs) as an estimate of the relative risk with GERD status as an independent variable and dietary factors as the dependent variable [29]. We evaluated the following additional variables as potential confounders: race/ethnicity (classified as white vs. non-white due to small sample sizes in the race/ethnic subgroups), smoking (ever vs. never and current vs. never), body mass index (BMI, kg/m^2^), medical center, recent alcohol use (number of drinks per week), aspirin or nonsteroidal anti-inflammatory drug (NSAID) use, a comorbidity index (the DxCg score) [30,31], education, income, serum *Helicobacter Pylori* (*H. Pylori*) antibody status, and energy consumption (kcal/day). Confounders were included in the final model if their inclusion altered the odds ratio by >10% [29]. We evaluated for interaction by gender, race, long-term (2 + years) vitamin supplement use, NSAIDs use, H. pylori, or regular use (at least once a week) of GERD medication (antacid, H2 blocker, or PPI) using cross product terms in the logistic regression model and stratified analysis. All analyses were performed using STATA statistical software (College Station, TX).

## Results

### Study population

Of the 316 GERD patients and 317 population controls from the original study, dietary data were available for 622 subjects (98% of interviewed subjects). Exclusion of six subjects with extreme values of caloric intake or ≥6 missing values on the food questionnaire and 126 population controls who reported acid regurgitation or heartburn more than once per month provided 308 GERD patients and 182 GERD-free population controls (Tables 1 and 2). The proportions of smokers and individuals who consumed large or extra-large portion sizes were similar between the groups.

**Table 1:** Characteristics of study groups

	**GERD Group (physician-assigned)**	**Population controls without GERD**^**1**^
	**Number or Mean (% or standard deviation)**	**Number or Mean (% or standard deviation)**
Number of subjects	308	182
Age (years)		
20-39	11 (4)	9 (5)
40-59	111 (36)	63 (35)
60-79	186 (60)	110 (60)
Race/Ethnicity		
White	247 (80)	154 (84)
Black	20 (6)	10 (5)
Hispanic	20 (6)	7 (4)
Asian	8 (3)	5 (3)
Others/Missing/Unknown	13 (4)	6 (3)
Male	211 (69)	123 (68)
GERD symptoms		
Severity^2^		
Severe & very severe	124 (40)	--
Moderate	109 (35)	--
Mild	75 (25)	--
Frequency		
Daily	78 (25)	--
Several times a week	89 (29)	--
Once month – once a week	91 (30)	--
Less than once a month	50 (16)	--
BMI	28.9 (5.2)	28.3 (5.5)
Smoking		
Current smoker	30 (10)	19 (10)
Portion size		
% extra large or large	27.9 (16.3)	28.8 (17.2)

^1^Self-reported heartburn or acid reflux <1/month.

^2^Severity with at least monthly or less than once a month frequency.

**Table 2:** Mean intake between physician-assigned GERD patients and population controls with no or little reported symptoms

	**GERD Group (physician-assigned)**	**Population controls without GERD**^**1**^
	**Number or Mean (% or standard deviation)**	**Number or Mean (% or standard deviation)**
Number of subjects	308	182
**Beverages**		
Soft drinks (servings/week)	3.2 (6.7)	2.1 (5.4)
Coffee (cups/day)	8.7 (10.4)	9.9 (9.9)
Tea (cups/day)	4.1 (7.4)	2.9 (5.2)
Alcohol (drinks/day)		
Non drinker	109 (35)	41 (23)
<=1/day	128 (42)	91 (50)
>1 & <2/day	29 (9)	31 (17)
> = 2/day	42 (14)	19 (10)
**Nutrients/food items**		
Calories (kcal/day)	1828 (770)	1797 (776)
Total fat (g/day)	77.9 (37.9)	77.5 (39.6)
Fried foods (fried potatoes, chickens, fish, doughnuts-servings/week)	0.7 (1.5)	0.6 (1.1)
Tomato products (servings/week)	1.5 (2.8)	1.5 (2.3)
Citrus (citrus fruits + juice- servings/week)	2.3 (3.8)	2.8 (3.5)

^1^Self-reported heartburn or acid reflux <1/month.

### Beverage choice and gastroesophageal reflux symptoms

Patients with moderate to severe GERD symptoms were over twice as likely as GERD-free controls to consume soft drinks, adjusting for smoking and education [OR = 2.11, 95% CI:1.16-3.84] (Table 3); there was no association with GERD frequency (Table 4).

**Table 3:** Association between moderate to severe GERD symptoms and intake of certain food items, comparing patients with moderate to severe symptoms to GERD-free population controls

**Severity**	**Moderate to severe GERD symptoms**				
	**N**		**OR**^**1**^	**95% CI**	
	**Case**	**Control**			
Softdrinks^2^					
None	70	86	1.0 (ref)		
>1/week	84	57	1.86	1.16	2.97
Coffee^3^					
None	92	55	1.0 (ref)		
2+/day	72	44	0.89	0.52	1.51
Tea^4^					
None	44	61	1.0 (ref)		
1+/day	51	41	1.86	1.02	3.40
Alcohol^5^					
None	116	57	1.0 (ref)		
<1 drink/day	74	70	0.56	0.35	0.90
1+ drink/day	48	70	0.83	0.46	1.48
Wine					
None	72	86	1.0 (ref)		
1 + glass/day	49	94	0.74	0.45	1.23
Beer					
None	53	99	1.0 (ref)		
1 + drink/day	29	101	0.54	0.31	0.96
Liquor					
None	31	126	1.0 (ref)		
1 + shot/day	36	99	1.50	0.85	2.66
Citrus					
<2servings/week	106	125	1.0 (ref)		
2+/week	59	133	0.62	0.41	0.94
Tomatoes					
None	55	176	1.0 (ref)		
1+/week	48	144	1.10	0.70	1.75
Fried foods					
<1serving/week	42	189	1.0 (ref)		
1+	49	143	1.52	0.94	2.45
Total fat					
1st Tertile (<57 g/day)	67	88	1.0 (ref)		
2nd Tertile	50	76	1.67	1.03	2.71
3rd T >90 g/day	66	52	1.77	1.07	2.93
Portion size					
<20% large portion	58	83	1.0 (ref)		
40 +%	55	56	1.30	0.78	2.19

^1^Models are adjusted for smoking and education.

^2^Not including diet soda.

^3^Includes both caffeinated and decaffeinated types.

^4^Excludes herbal tea.

^5^Excludes individuals who are occasional drinkers (more than once a year but less than one drink/week).

**Table 4:** Association between the frequency of GERD symptoms and intake of certain food items (GERD patients vs. no-GERD population controls)

**Frequency**	**> = Several times/week**					**Once a month to once a week**				
	**N**		**OR**^**1**^	**95% CI**		**N**		**OR**^**1**^	**95% CI**	
	**Case**	**Control**				**Case**	**Control**			
Softdrinks^2^										
None	46	50	1.0 (ref)			46	50	1.0 (ref)		
>1/week	97	70	1.54	0.93	2.58	31	47	0.75	0.41	1.39
Coffee^3^										
None	56	37	1.0 (ref)			56	37	1.0 (ref)		
2+/day	83	53	0.98	0.56	1.72	47	27	1.25	0.65	2.39
Tea^4^										
None	29	36	1.0 (ref)			29	36	1.0 (ref)		
1+/day	57	50	1.54	0.81	2.93	20	37	0.70	0.34	1.47
Alcohol^5^										
None	72	31	1.0 (ref)			72	31	1.0 (ref)		
<1 drink/day	91	82	0.51	0.30	0.85	56	36	0.69	0.38	1.26
1+ drink/day	53	82	0.68	0.35	1.30	30	37	0.88	0.42	1.85
Wine										
None	48	51				48	51	1.0 (ref)		
1 + glass/day	56	111	0.64	0.37	1.10	36	47	1.02	0.54	1.91
Beer										
None	31	57	1.0 (ref)			31	57	1.0 (ref)		
1 + drink/day	35	120	0.52	0.28	0.96	28	59	0.89	0.46	1.71
Liquor										
None	19	74	1.0 (ref)			19	74	1.0 (ref)		
1 + shot/day	43	120	1.44	0.77	2.70	17	70	1.07	0.51	2.26
Citrus										
<2servings/week	63	78	1.0 (ref)			63	78	1.0 (ref)		
2+/week	69	118	0.62	0.40	0.98	55	132	1.03	0.62	1.69
Tomatoes										
None	28	113	1.0 (ref)			28	113	1.0 (ref)		
1+/week	55	171	1.35	0.80	2.28	31	91	1.44	0.80	2.59
Fried foods										
<1serving/week	20	121				20	121	1.0 (ref)		
1+	59	167	2.10	1.19	3.70	29	93	1.87	1.00	3.52
Total fat										
1st Tertile (<57 g/day)	48	57	1.0 (ref)			48	57	1.0 (ref)		
2nd Tertile	88	67	1.57	0.94	2.62	40	38	1.27	0.70	2.31
3rd T >90 g/day	71	70	1.69	0.98	2.91	44	40	1.89	1.03	3.47
Portion size										
<20% large portion	33	52	1.0 (ref)			33	52	1.0 (ref)		
40 +%	61	71	1.23	0.75	2.02	33	43	1.01	0.57	1.77

^1^Models are adjusted for smoking and education.

^2^Not including diet soda.

^3^Includes both caffeinated and decaffeinated types.

^4^Excludes herbal tea.

^5^Excludes individuals who are occasional drinkers (more than once a year but less than one drink/week).

Patients with moderate to severe GERD symptoms were also more likely to consume one or more cups of regular tea a day compared to asymptomatic controls [OR = 1.86; 95% CI:1.16-2.97 for severe GERD]. There was no association between GERD symptom severity or frequency and coffee consumption.

GERD patients with severe or frequent symptoms were less likely to consume beer, and there was also an inverse association between GERD symptom severity and general alcohol drinking, especially light drinking. For instance, patients with moderate to severe symptoms were almost half as likely to drink beer as those without GERD [OR = 0.54 95% CI 0.31-0.96]. Similarly, patients with frequent GERD symptoms (>1/week) were half as likely to drink beer [OR = 0.52 95% CI: 0.28-0.96]. There were non-significant, though positive association between GERD symptom severity and frequency and liquor drinking: individuals with severe GERD symptoms were 50% more likely to drink liquor compared to those without GERD [OR = 1.50 95% CI: 0.85-2.66].

### Food, total fat, portion size and gastroesophageal reflux symptoms

Patients with severe or frequent GERD symptoms were less likely to consume citrus fruits compared to asymptomatic controls [OR = 0.62; 95% CI: 0.41-0.94; OR = 0.62; 95% CI: 0.40-0.98, respectively]. On the other hand, there was no association between the severity or frequency of GERD symptoms and consumption of tomato products, another acidic food which GERD patients are recommended to avoid (Tables 3 and 4).

Individuals with severe symptoms had higher total fat consumptions than those without GERD [OR = 1.77 95% CI: 1.07-2.93 for highest tertile; OR = 1.67 95% CI: 1.03-2.71 for second tertile]. Similar associations were observed for frequency of GERD symptoms and total fat. In addition, individuals with frequent symptoms were twice as likely to consume fried foods [OR = 2.10; 95% CI: 1.19-3.70 for most frequent; OR = 1.87 95% CI: 1.00-3.52 for monthly-weekly frequency]. There was no association between the severity or frequency of GERD symptoms and large portion sizes.

### Supplementary analyses

#### *Composite severity and frequency GERD and diet*

To evaluate the dietary habits of individuals with severe *and* frequent GERD symptoms, we created a composite variable including those with moderate to severe symptoms with at least weekly frequency and compared with asymptomatic controls. Most of the individuals who were in the moderate to severe category overlapped with this composite category (120/124 = 97%), and the results were similar to those presented in Table 3, though the associations strengthened slightly. For instance, individuals with severe and frequent symptoms were twice as likely as controls to consume soft drinks or tea [OR = 1.98 95% CI: 1.18-3.31; OR = 2.16 95% CI: 1.15-4.03, respectively].

#### *Effect modification*

The association between soft drink consumption and GERD severity was stronger among women [OR = 5.71 95% CI 1.75-18.66] than among men [OR = 1.55 95% CI: 0.75-3.23].

### Analysis of potential confounding

There was no evidence of strong confounding by age, gender, ethnicity, BMI, NSAIDS, alcohol use, income, total energy, facility of diagnosis, income, co-morbidity score, long-term vitamin supplement use, or serum H. *pylori* antibody status. A fully adjusted model for citrus intake vs. moderate to severe GERD symptoms (containing all these factors plus education and smoking), for example, produced effect estimates [OR = 0.56; 95% CI: 0.29-0.77] similar to a model that contained only education and smoking [OR = 0.62; 95% CI: 0.41-0.94]. The results were similar for other dietary variables.

## Discussion

The current study, to our knowledge, is the first population or community-based study to assess whether patients with GERD adhere to dietary guidelines that are often recommended as a part of non-pharmacological, lifestyle modification to reduce their symptoms [17,18]. Patients with severe or frequent GERD symptoms were less likely to consume citrus and beer and more likely to consume tea, soft drinks, total fat, and possibly liquor. There was no association between portion size, tomato products, coffee and GERD severity or frequency.

Our study extends the existing knowledge regarding the relationship between GERD symptoms and dietary intake. First, our data demonstrate that patients with more severe or frequent reflux symptoms are less likely to consume citrus. Citrus and other acidic foods such as tomatoes are often considered reflux-triggering. A previous clinical study reported that acid-sensitive patients were sensitive to intraesophageal infusion of orange or tomato juice, even when the pH of juice was adjusted to neutral [32], suggesting that some other component in citrus or tomato beside acidity may affect the symptoms. We cannot assess from our data whether the patients in our study avoided citrus (but not tomato products) because they actually worsened their symptoms or because of perceived stronger recommendations to avoid citrus than tomato products.

Our results suggest that individuals with severe GERD symptoms are less likely to drink beer but more likely to drink liquor. Alcohol ingestion is thought to induce GERD by reducing lower esophageal sphincter pressure, increasing acid secretion through gastrin stimulation, decreasing esophageal motility and impairing gastric emptying [33-36]. Multiple differences exist between beverage types including alcohol content (lower in beer), volume (higher per serving for beer), and carbonation (present in beer but not liquor). These differences may partially explain the alcohol choices found in GERD patients, although we cannot exclude confounding by other factors, since liquor drinkers may differ from beer drinkers in many ways.

Patients with moderate to severe GERD symptoms were more likely to drink soft drinks than controls. Soft drinks, often carbonated, may result in a very short decline in intra-esophageal pH and transient reduction in lower esophageal sphincter basal pressure [37]. A recent small clinical experiment demonstrated that in healthy individuals, ingestion of carbonated water, caffeinated cola, or caffeine-free cola reduced lower esophageal sphincter pressure compared with tap water ingestion [38]. A multicenter, longitudinal, cohort study of the cardiovascular consequences of sleep-disordered breathing reported that carbonated soft drink consumption was a major predictor of nocturnal heartburn [39]. Our data suggests that, despite the recommendation to avoid soda, GERD patients do not avoid its consumption.

This study has potential limitations. First, cross-sectional studies cannot establish cause and effect [29]. The inverse associations, such as the avoidance of citrus among GERD subjects, could be because such foods cause GERD symptoms (so the subject avoids them), because patients adhere to the dietary guidelines even if avoidance is not completely ineffective, or even conceivably because the absence of such foods causes GERD (and the reason the person has GERD is because they don’t consume them), although the latter possibility seems less likely for the foods studied. In contrast, the positive association between soda consumption and GERD symptoms, for example, could be either from soft drinks causing the greater GERD symptoms or, alternatively, patients actually drinking soda to relieve their GERD symptoms, despite the reported adverse mechanistic associations. However, if the association is true (GERD patients drink more soft drinks than controls and soft drinks worsen symptoms), then it may indicate that GERD patients may benefit from enhanced dietary modification. Second, the GERD patients evaluated, by study definition, had a physician-assigned diagnosis of GERD and had previously received anti-secretory medications. This population may represent patients with more severe GERD symptoms than a general population sample; however, it also represents patients who, if medications have potential for harm, may benefit most from nonpharmacologic treatments (if they are effective). Third, some of the dietary information obtained for this study was not complete. For instance, we did not have information on types of coffee (decaffeinated or regular).

Strengths of the present study include its community-based design, random sampling of patients from the population base (rather than convenience sampling of clinic-based patients), use of a validated food frequency questionnaire and a validated GERD questionnaires for symptom frequency and intensity, in person interviews for GERD symptoms, the availability of high quality interview and laboratory data for multiple confounders, and the review of pertinent medical records. In addition, we used a well characterized population of GERD patients confirmed by both self-report and a physician diagnosis. The combination of these features provides a high quality evaluation of the associations within the limits of an observational study. Subjects came from a diverse KPNC membership base that closely approximates the region’s census demographics, and the results can likely be generalized to similar large populations.

## Conclusions

In summary, to our knowledge, this is the first large, community-based U.S. population to evaluate whether GERD patients adhere to the dietary guidelines commonly recommended for GERD patients. We found that while GERD patients, consistent with dietary recommendations, are less likely to consume citrus and certain types of alcohol, they were more likely to consume soft drinks, tea, and fatty foods than asymptomatic controls. The results suggest that GERD patients do avoid some, but not all, of the foods commonly thought to worsen GERD symptoms. Given the prevalence of GERD symptoms and the emergence of possible adverse effects of long-term gastric acid inhibition, more studies, particularly randomized clinical trials on the effectiveness of dietary modification, are needed to better understand the role of nonpharmacologic approaches, such as dietary modification, in the treatment of GERD.

## Competing interests

The authors declare that they have no competing interests.

## Authors’ contributions

AK participated in the data analysis, interpretation of results, and manuscript writing. GB, and PB participated in acquisition of data, interpretation of results and editing the manuscript. CQ participated in interpretation of the results and editing the manuscript. DC conceived of the study and participated in the study design, acquisition of data, interpretation of results, and editing the manuscript. All authors read and approved the final manuscript.

## Pre-publication history

The pre-publication history for this paper can be accessed here:

http://www.biomedcentral.com/1471-230X/14/144/prepub
